# Identification and functional analysis of endogenous nitric oxide in a filamentous fungus

**DOI:** 10.1038/srep30037

**Published:** 2016-07-18

**Authors:** Anchalee Pengkit, Seong Sil Jeon, Soo Ji Son, Jae Ho Shin, Ku Yeon Baik, Eun Ha Choi, Gyungsoon Park

**Affiliations:** 1 Plasma Bioscience Research Center, Kwangwoon University, Seoul, 01897, Republic of Korea; 2 Department of Electrical and Biological Physics, Kwangwoon University, Seoul, 01897, Republic of Korea; 3 Department of Chemistry, Kwangwoon University, Seoul, 01897, Republic of Korea

## Abstract

In spite of its prevalence in animals and plants, endogenous nitric oxide (NO) has been rarely reported in fungi. We present here our observations on production of intracellular NO and its possible roles during development of *Neurospora crassa*, a model filamentous fungus. Intracellular NO was detected in hypha 8–16 hours after incubation in Vogel’s minimal liquid media and conidiophores during conidiation using a fluorescent indicator (DAF-FM diacetate). Treatment with cPTIO, an NO scavenger, significantly reduced fluorescence levels and hindered hyphal growth in liquid media and conidiation, whereas exogenous NO enhanced hyphal extension on VM agar media and conidia formation. NO scavenging also dramatically diminished transcription of *con-10* and *con-13,* genes preferentially expressed during conidiation. Our results suggest that intracellular NO is generated in young hypha growing in submerged culture and during conidia development and regulate mycelial development and conidia formation.

Microorganisms endogenously produce reactive oxygen and nitrogen species, although reactive species have anti-microbial effects^1^,^2^,^3^. Indeed, regulatory roles of reactive oxygen species such as superoxide (O_2·_^−^) and hydrogen peroxide (H_2_O_2_) in cell differentiation have been demonstrated in many microorganisms^4^,^5^,^6^. Comparably, reactive nitrogen species in microbial cells have been explored infrequently, but their involvement in a wide variety of cellular functions has been recently unveiled^7^,^8^,^9^. Studies show that nitric oxide (NO), known as a ubiquitous signaling molecule in mammalian and plant cells, can be endogenously synthesized and is involved in the regulation of many processes in microbial cells^8^,^9^,^10^,^11^,^12^,^13^,^14^,^15^. Although NO activities identified in microbial cells are somewhat similar to those found in animals and plants, differences in origin and functions are also known.

In many bacteria, NO is synthesized from L-arginine and oxygen by the catalytic activity of nitric oxide synthases (NOSs), which are structurally different from mammalian NOSs^14^,^16^. Mammalian NOS has three isoforms, neuronal NOS (nNOS), inducible NOS (iNOS), and endothelial NOS (eNOS), and all forms have N-terminal catalytic oxygenase domains and C-terminal reductase domains^17^. In contrast, most bacterial NOSs are structurally simple consisting of an oxygenase domain, with some exceptions possessing additional domains^18^. While endogenously produced NO in mammals and plants is involved in neurotransmission, vasodilation, and regulation of abiotic and biotic stresses, NO synthesized by bacterial NOS plays roles in alleviation of oxidative stress and UV damage, transcriptional regulation, and biosynthesis of nitrated compounds^16^,^19^,^20^.

Compared to bacteria, the biosynthesis and functions of endogenous NO in fungi have not been thoroughly explored. Using NO donors, mammalian NOS inhibitors, and NO scavengers, production and cellular functions of NO were indirectly demonstrated in fungi such as *Blastocladiella emersonii*, *Candida tropicalis*, *Collectotrichum coccodes*, *Coniothyrium minitans*, *Flammulina velutipes*, *Neurospora crassa*, *Phycomyces blakesleeanus*, *Physarum polycephalum*, and *Saccharomyces cerevisiae*^7^,^9^,^10^,^12^,^21^,^22^,^23^,^24^,^25^. Recently, intracellular NO has been directly detected using a fluorescent indicator in several fungi such as *Aspergillus nidulans* and *Magnaporthe oryzae*^8^,^26^,^27^. In these fungi, NO regulates conidiation, fruiting body formation, pseudomycelial formation, conidial germination, and infection. Compared to mammals, plants, and bacteria, NO in fungi shared similar roles during cell differentiation but also possess distinct functions in fungal life. Particularly, synthesis of NO in fungi does not seem to follow NOS dependent pathways, as shown in animals, plants, and bacteria. Despite the discovery of NOS like activities in several fungi, evidence for fungal NOS is still limited^8^,^12^,^25^,^28^.

As eukaryotic microorganisms, fungi share many common cellular processes with other eukaryotic organisms, but NO biology in fungi seems to be different in terms of mode of production and biological functions. In addition, evidence for the direct detection of NO in fungal cells is few, and the origin of intracellular NO and its functions are still unknown in many fungi. In this study, we analyzed intracellular production and functions of NO during fungal development using a model filamentous fungus, *N. crassa*. Recent work in *N. crassa* indirectly demonstrated the presence of NO and showed that NOS inhibitors enhanced conidiation and exogenous NO inhibited light induced conidiation^21^. However, the majority of NO biology in *N. crassa* is still unknown due to lack of direct detection of intracellular NO and lack of NOS like genes in the *N. crassa* genome. Therefore, our study was designed to elucidate endogenous generation and biological functions of NO in *N. crassa*.

## Results

### Intracellular NO is elevated during hyphal growth and conidia formation

**Figure 1:**
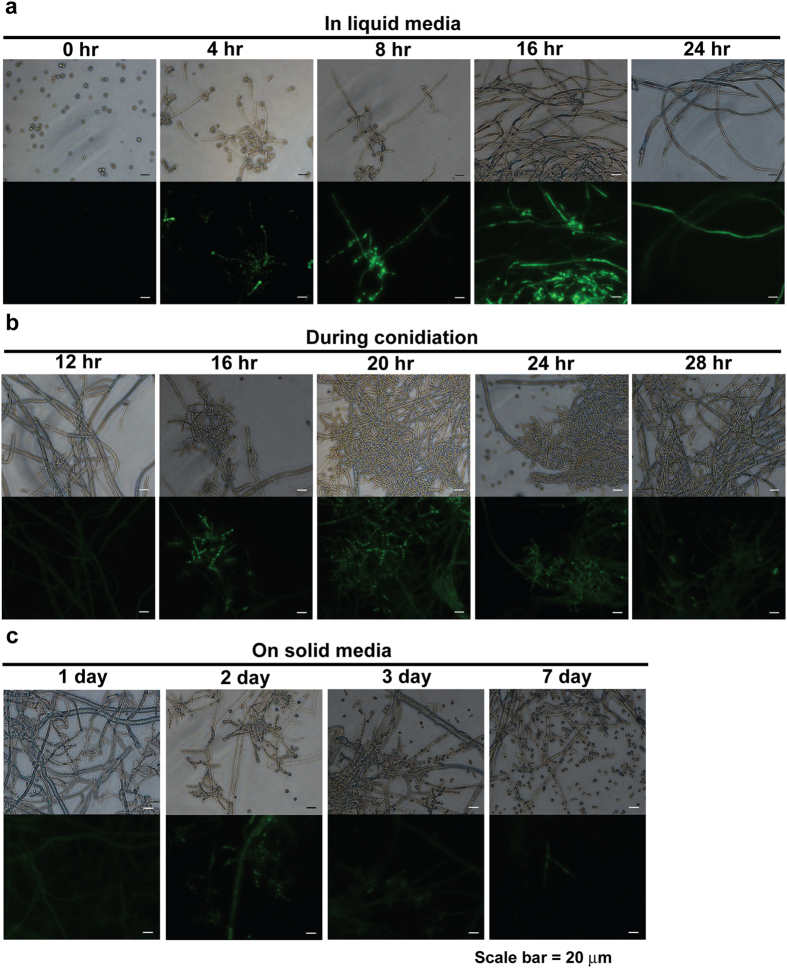
NO production during fungal development. Intracellular NO detected using DAF-FM diacetate during (**a**) vegetative growth in VM liquid, (**b**) conidiation on VM agar media, and (**c**) on VM agar media. Top and bottom of each picture indicate images captured in optical and fluorescent (465–495 nm excitation filter) mode, respectively.

Intracellular NO levels were measured using a cell permeable fluorescent indicator, DAF-FM diacetate. After DAF-FM diacetate enters a cell, the acetyl group is hydrolyzed by intracellular esterase and DAF-FM, which is not fluorescent, becomes benzotriazole and fluoresces when it reacts with NO^29^. Fluorescent intensity therefore indicates the level of intracellular NO. Using this method, fluorescence detected by DAF-FM diacetate was increased in fungal hypha grown for 8–16 hours in VM (Vogel’s Minimal) liquid (Fig. 1a) and in conidiophores after 16–20 hours on VM agar plates, used to induce synchronized aerial hypha formation and conidiophore development (Fig. 1b). When spores were inoculated on VM agar plates and incubated, higher NO levels were observed in a 2 day-old culture (Fig. 1c). Development of conidiophores was more prominent after 2 days on VM agar plates, and the majority of fluorescence appeared in conidiophores (Fig. 1c).

**Figure 2:**
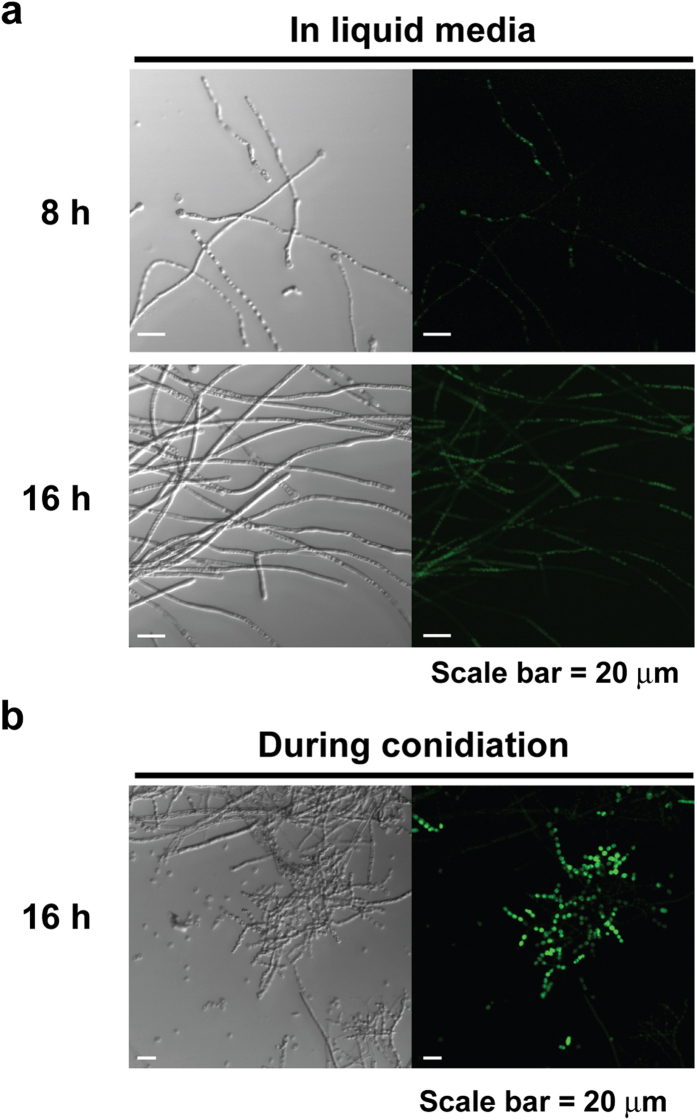
Intracellular NO analyzed by confocal microscopy. Intracellular NO detected by DAF-FM diacetate was observed in fungal hypha grown in VM liquid for (**a**) 8 and 16 hours and (**b**) in fungus during conidiation. Left and right of each picture indicate images captured in optical and fluorescent (465–495 nm excitation filter) mode, respectively.

To identify the location of intracellular NO, fungal samples were further analyzed using confocal microscopy. This analysis showed that high fluorescence detected in 8 and 16 hour VM liquid cultures was mostly in vacuole like structures (Fig. 2a). In conidiation on VM agar media incubated for 16 hours, areas containing conidiophores were highly fluorescent compared to hypha (Fig. 2b).

**Figure 3:**
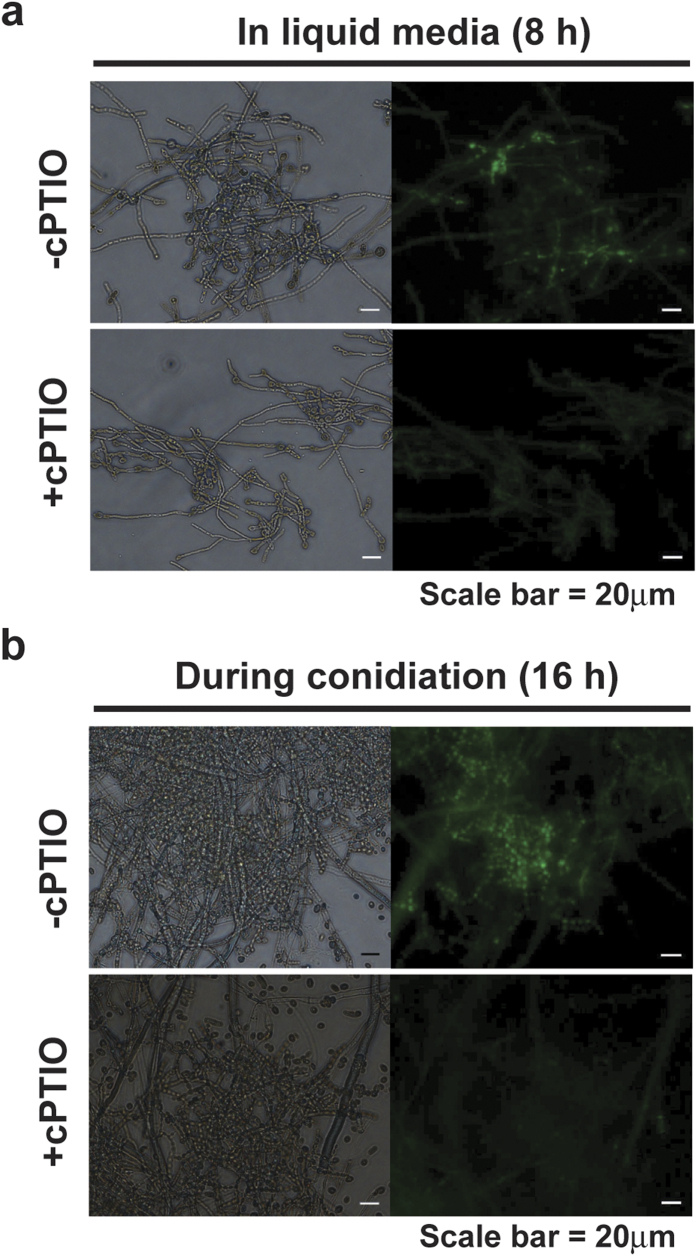
Effects of cPTIO on intracellular NO levels. (**a**) Intracellular NO detected by DAF-FM diacetate in hypha grown for 8 hours in VM liquid after treatment with or without 10 mM cPTIO for 20 minutes. (**b**) Intracellular NO in fungus incubated for 16 hours after conidiation was induced, with or without 10 mM cPTIO. Left and right of each picture indicate images captured in optical and fluorescent (465–495 nm excitation filter) mode, respectively.

We treated fungi with an NO scavenger, cPTIO (2-(4-carboxyphenyl)-4,5-dihydro-4,4,5,5-tetramethy-1 H-imidazolyl-1-oxy-3-oxide), to confirm that fluorescence measured by DAF-FM was due to NO. As shown in Fig. 3a, bright fluorescence was exhibited in fungal hypha grown in VM liquid for 8 hours, whereas much lower levels of fluorescence were detected in fungal hypha after treatment with cPTIO (Fig. 3a). Fluorescence observed in conidiophores after 16 hours in conidiation induction culture was also significantly decreased after cPTIO treatment (Fig. 3b). These results show that removal of NO by cPTIO causes reduced fluorescence, therefore indicating the presence of NO.

### NO scavenger treatment hinders hyphal growth and slows conidiation

**Figure 4:**
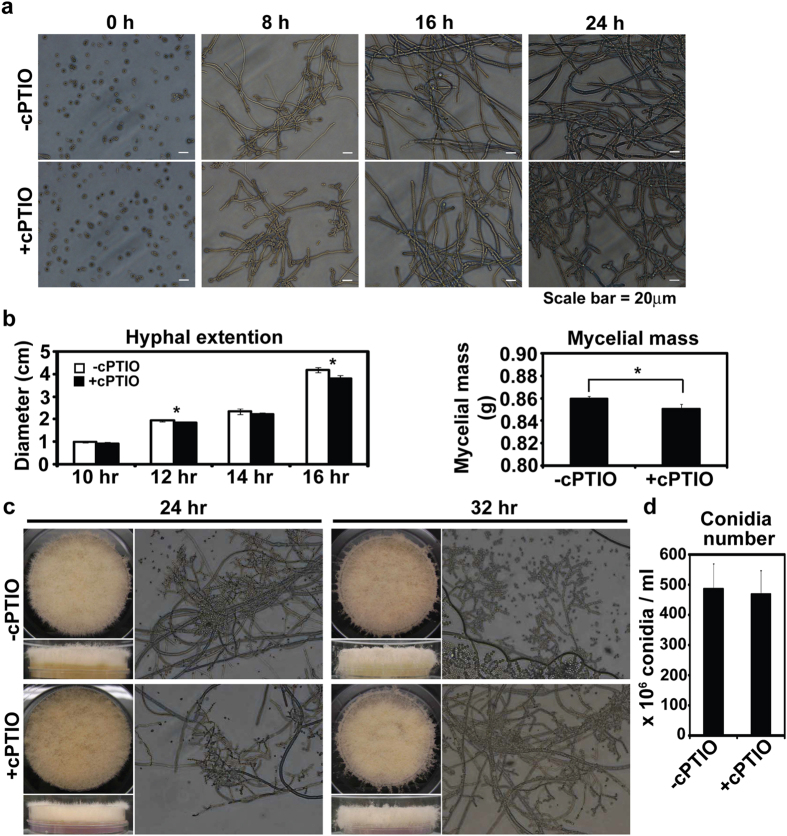
Effects of cPTIO on fungal development. (**a**) Hyphal growth in VM liquid with or without 10 mM cPTIO. (**b**) Hyphal extension on VM agar media (left panel) and weight of mycelia grown in VM liquid (right panel) in the presence of cPTIO. All values represent mean ± standard deviation of 3 replicate measurements. (**c**) Conidiation synchronously induced on VM agar media after treatment with or without cPTIO. (**d**) Number of conidia collected from 5 day-old cultures after cPTIO treatment. All values represent mean ± standard deviation of 3 replicate measurements.

Because intracellular NO was increased in fungal hypha and conidiophores, we hypothesized that NO plays a role in regulating hyphal development and conidiation. To test this hypothesis, fungal development was monitored after NO was removed using an NO scavenger. In VM liquid culture, the presence of cPTIO modestly inhibited hyphal branching as observed under microscope (Fig. 4a). When cPTIO was added to VM agar plates (60 mm in diameter), basal hyphal extensions were significantly reduced compared to control after 16 hours (Fig. 4b). The dry weight of hyphal mass collected after 24 hours was also significantly smaller in cPTIO treated samples than control (Fig. 4b). On cPTIO treated VM agar, development of conidiophores was delayed compared to control (Fig. 4c), but aerial hyphal development of mycelia was not different. More conidiophores were observed on control than cPTIO treated VM agar plates after 24 hours (Fig. 4c), and, after 32 hours, conidia were released from conidiophores in control while cPTIO treated conidiophores were still holding conidia (Fig. 4c). Conidia were harvested after 5 days, revealing that the number of conidia per ml suspension was slightly decreased in cPTIO treated samples compared to control, although the difference was not significant (Fig. 4d).

### NO scavenger treatment decreases transcription of conidiation related genes

**Figure 5:**
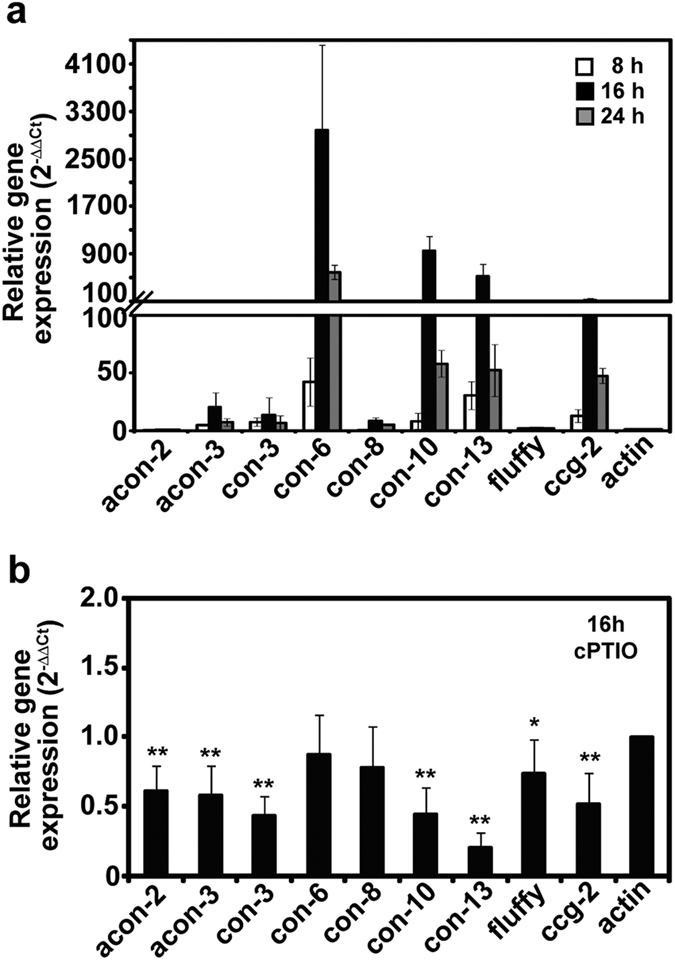
Transcription of *con* genes during conidiation after cPTIO treatment. (**a**) Transcript levels of *con* genes in wild type *N. crassa* after synchronous conidiation was induced. All values are mean ± standard deviation of 6 replicate measurements from 2 independent experiments. (**b**) c*on* gene transcripts 16 hours after conidiation was initiated in wild type treated with cPTIO. Student t-test was performed between control and each treatment; *p < 0.05, **p < 0.01.

During development of aerial hypha and conidiophores from fungal mycelia placed on VM agar, we observed that transcription of several *con* genes (known as preferentially expressed during conidiation) was increased (Fig. 5a). Among 9 putative genes involved in the regulation of conidiation (*acon-2*, *acon-3*, *con-3*, *con-6*, *con-8*, *con-10*, *con-13*, *fluffy*, *ccg-2*), the level of *con-6*, *con-10*, *con-13*, and *ccg-2* transcripts was significantly increased compared to the other genes 16 hours after conidiation was induced (Fig. 5a). After 24 hours, transcript levels of these 4 genes decreased, although levels were still higher than those of other genes (Fig. 5a). When fungus was treated with the NO scavenger, cPTIO, a significant reduction in transcription of *con-10*, *con-13*, and *ccg-2* was observed 16 hours after conidiation was induced compared to the control (Fig. 5b). Particularly, transcript level of *con-13* after cPTIO treatment was much lower than that of control (Fig. 5b). These results indicate that removal of NO by cPTIO suppressed the expression of these 3 genes that are specifically associated with conidial development and maturation.

### Basal hyphal growth and conidia formation are increased by exogenous NO

**Figure 6:**
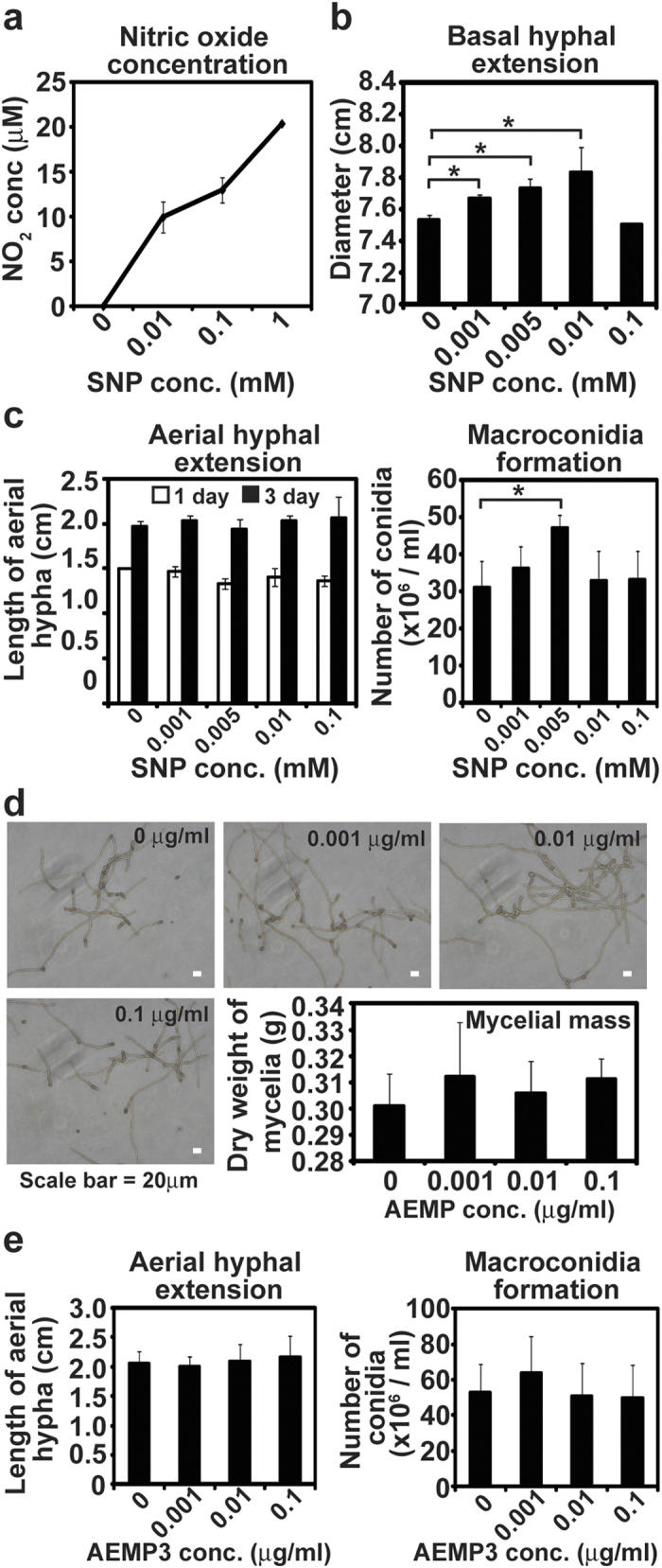
Vegetative growth and conidiation in *N. crassa* treated with SNP and AEMP3. (**a**) NO released in different concentrations of SNP solution. All measurements were performed in 3 replicates. (**b**) Extension of basal hyphae on VM agar media supplemented with SNP. (**c**) Aerial hyphal extension (left panel) on the surface of VM liquid containing SNP and number of conidia (right panel) harvested from the culture after 5 days. All values are mean ± standard deviation of 3 replicate measurements and the experiments were repeated 3 times. Student t-test was performed between control and each treatment; *p < 0.05. (**d**) Hyphal development after 8 hours incubation in VM liquid supplemented with NO releasing nanoparticle AEMP3 (microscopic photographs). After 3 days, dry weight of mycelia mass harvested (graph). (**e**) Aerial hyphal extension (left panel) on the surface of VM liquid containing different concentrations of AEMP3 and number of conidia (right panel) harvested from cultures after 5 days. All values are mean ± standard deviation of 3 replicate measurements and the experiments were repeated 3 times.

Since scavenging intracellular NO by cPTIO caused retardation in vegetative hyphal growth and conidial development, addition of exogenous NO may also influence these developmental processes. We treated wild type *N. crassa* with exogenous NO using SNP (sodium nitroprusside), an NO releasing chemical, and AEMP3, an NO releasing nanoparticle. SNP is a well known NO producing chemical, and NO concentrations in SNP solution, measured as nitrite (NO_2_^−^) level, are shown in Fig. 6a. NO levels increased linearly as SNP concentration increased (Fig. 6a). NO reached a concentration of 20 μM in 1 mM SNP solution (Fig. 6a).

After 24 hours of incubation, basal hyphal extension significantly increased on VM agar media when SNP was added (Fig. 6b). The diameter of hyphal extensions was also greatest in media with 0.01 mM SNP (about 10 μM NO), whereas there was no dramatic change in hyphal extension in the presence of 1 mM SNP, compared to control (Fig. 6b). During conidial development, addition of SNP did not affect aerial hyphal growth, although the number of conidia, after a 5 day incubation, was significantly higher in the presence of 0.005 mM SNP (about 5 μM NO) compared to control (Fig. 6c).

Nitric oxide-releasing AEMP3 silica nanoparticles were employed as another NO donor^30^. The NO release properties were characterized in phosphate buffered saline (PBS; 0.01 M, pH 7.4) at 37 °C as follows (see Supplementary Fig. S1). The total amount of NO released (t[NO]) and maximum NO flux ([NO]_m_) from the AEMP3 nanoparticle system were 0.59 μmol·mg^−1^ and 1180 ppb·mg^−1^, respectively. The NO release kinetics from the AEMP3 silica nanoparticles were relatively slow compared to other NO-releasing silica nanoparticle systems^30^ with a NO release half-life (*t*_1/2_) of 130 minutes. In addition, the time required to reach the maximum NO flux (*t*_m_) of 1180 ppb·mg^−1^ was determined by 5 min after immersion in buffer solution. As characterized by scanning electron microscopy (SEM), the size of the 40 mol% AEMP3 nanoparticles was 23 ± 2 nm. When NO releasing AEMP3 nanoparticles were added in VM liquid, fungal hyphal growth and asexual development (aerial hyphal development and conidia formation) were not significantly changed. However, slight enhancement was observed in hyphal mass and conidia number after treatment with AEMP3 in every repeated experiment (Fig. 6d,e). The dry weight of fungal mycelia grown for 3 days in the presence of AEMP3 was slightly higher than that of control (Fig. 6d). The height of aerial hypha developed on the surface of VM liquid was not different between control and AEMP3 treated samples, whereas the number of conidia collected after 5 days was slightly greater in the culture supplemented with 0.001 μg/ml AEMP3 (Fig. 6e).

### NO level is not changed in knockout mutants of NOS like genes in *N. crassa*

To find the mechanism(s) for NO synthesis, we first searched candidate genes homologous to NOS (nitric oxide synthase) from other organisms in *N. crassa* genome. Several *Neurospora* genes showing high homology to NOSs of human^17^ and other fungi^8^,^9^,^11^ (*M. oryzae*, *P. polycephalum*, *I. obliquus*) were identified (Table 1). Homology of these genes to human NOSs was relatively lower than that to fungal NOSs (Table 1). Among candidate genes, NCU05006, NCU05185, and NCU09741 appeared to be highly homologous to human and other fungal NOSs (lowest expected value) (Table 1).

**Figure 7:**
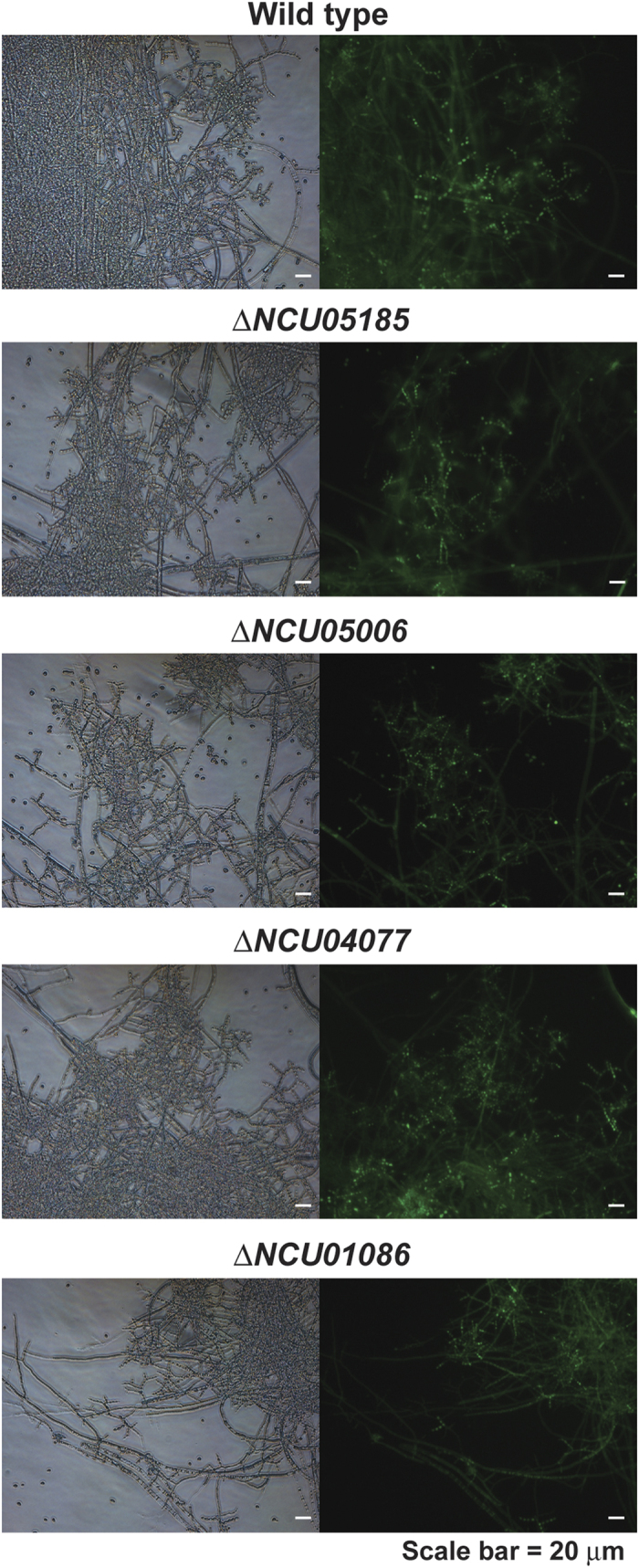
NO production in knockout mutants of NOS like genes in *N. crassa*. Intracellular NO was detected in wild type and knockout mutants of NCU05185, NCU05006, NCU04077, and NCU01086 homologous to NOS in human and other fungi. NO was detected using DAF-FM diacetate in fungal samples incubated for 16 hours after conidiation was induced. Left and right of each picture indicate images captured in optical and fluorescent (465–495 nm excitation filter) mode, respectively.

Intracellular NO was detected using DAF-FM diacetate in knockout mutants of these candidate genes. Since there were no available homokaryotic mutants for several genes including NCU09741 (probably essential gene for viability or sexual development), we examined NO in knockout mutants of only NCU01086, NCU04077, NCU05006, and NCU05185 grown for 16 hours after synchronous conidiation was induced. No dramatic difference in intracellular NO level was observed between wild type and mutants as fluorescence intensity in conidiophores was similar in all samples (Fig. 7). This indicates that these tested genes may not be the genes coding for NOS or NO synthesis in *N. crassa* does not follow NOS dependent way. Nevertheless, we still need to examine mutants of all candidate genes.

NCU01086, NCU04077, NCU05006, and NCU05185 seem to be dispensable to the phenotypes associated with intracellular NO level such as vegetative growth and conidiation in *N. crassa*. Knockout mutants of these genes did not show any significant changes in vegetative growth and conidiation, showing similar phenotypes to wild type (Supplementary Figs S2 and S3). It is likely that NCU01086, NCU04077, NCU05006, and NCU05185 are not much involved in NO synthesis and related pathways although further study is necessary.

## Discussion

Endogenous generation of reactive species in microbial cells is an intriguing process, because reactive species have antimicrobial activities. However, increasing numbers of studies demonstrate that intracellular reactive species play critical roles in microbial vitality, including management of stresses and differentiation^2^,^14^. Among all reactive species, NO has been considered a universal signaling molecule in most organisms including microorganisms. However, very little is known about the synthesis and functions of NO in fungi. In most fungi, endogenous NO generation has been indirectly verified by identifying NOS-like activity or using NO scavengers^7^,^10^,^12^,^21^,^22^,^23^,^24^,^25^. Few studies have demonstrated direct detection of intracellular NO^8^,^26^,^27^.

In our study, endogenous NO in *N. crassa* was directly detected using the NO indicator, DAF-FM diacetate. Our data clearly show that NO is generated in cells during conidia formation on agar media and hyphal branching in liquid VM. Therefore, our data have provided direct evidence for endogenous NO production, which was not directly detected in Ninnemann and Maier’s study^21^. High fluorescence detected inside vacuole like structures in fungal hyphae may indicate the subcellular place of NO synthesis or be a result of accumulation of DAF-FM dye in vacuole. We also observed high DAF-FM fluorescence inside vacuolar structures in *Fusarium oxysporum* hyphae during sporulation in VM liquid (Supplementary Fig. S4). Intracellular NO level was increased during microconidia formation (16–24 h) in *F. oxysporum* but fluorescence was highly detected in vegetative hyphae not in phialides and microconidia (Supplementary Fig. S4). This result was different from that observed in *N. crassa* in which NO was detected highly in conidiophores during conidiation. In *Arabidopsis thaliana*, NO is detected inside the vacuole in immature growing root hairs and then the cytoplasm after maturation^31^. Vacuole is known as a storage reservoir for basic amino acids such as arginine in filamentous fungi^32^. It may be possible that NOS dependent NO synthesis can occur in vacuole but other possibilities should be also considered.

The origin of intracellular NO in *N. crassa* is still obscure. We identified several putative genes homologous to human and other fungal NOSs in the *N. crassa* genome (Table 1). None of the knockout mutants tested showed reduced NO levels and phenotypes during vegetative growth and conidiation. Since we did not examine mutants of all candidate genes, further analyses may be needed. Our results also suggest a possibility of more various routes for NO synthesis (NOS dependent and independent) in *Neurospora*, compared to animal. *Neurospora* NOSs may have a very different genome sequence or non-enzymatic synthesis can be possible. Both NOS dependent and independent pathways can be considered in *Neurospora* NO production as demonstrated in plants in which sequence of NOS gene is different from that of mammalian NOS^33^, and NO is also generated during nitrate reduction^34^, by the action of xanthine oxidase^35^, or by non-enzymatic ways^36^,^37^. It is recently suggested in *A. nidulans* that NO production can be catalyzed by nitrate reductase during development and more than one pathways can be involved in NO synthesis^27^. The implication here is that NO synthesis in fungi can be quite distinct from those found in other organisms. Our preliminary data show that deletion of a putative xanthine oxidase gene (NCU03350) does not impair NO synthesis during submerged hyphal development in *N. crassa* (Supplementary Fig. 5). We also did screening knockout mutants of transcription factors and serine/threonine protein kinases that exhibited defects in vegetative hyphal growth and conidiation for recovery of defects by exogenous NO (see Supplementary methods and Table S1). Aerial hyphal growth and conidia formation were partially recovered in knockout mutant of NCU05658 (*stk-36*) (Supplementary Table S2), a homolog of yeast SKY1 that is known to regulate polyamine transport and ion homeostasis^38^. Since exogenous polyamine is known to enable NO production in *A. thaliana*^39^, it may be possible that polyamine metabolism is related to NO synthesis in *N. crassa*. Further investigation will be needed on this.

Our data suggest several possible roles for NO in *Neurospora* development. First, because intracellular NO is detected in conidiophores and the NO scavenger cPTIO delays conidiophore development and reduces the number of conidia, NO may be involved in formation of macroconidia. Roles for NO in conidiation have been demonstrated in fungi, where production of NO is verified^11^,^12^,^21^,^23^. However, our results are somewhat contrary to the observation made in the Nimmemann and Maier’s study, in which inhibition of NOS activity enhanced conidiation and exogenous NO reduced conidiation^21^. This may be because homologous NOS genes in *N. crassa* do not seem to be related to NO generation, recall that use of a mammalian NOS inhibitor did not reduce NO levels. In addition, the concentration of SNP, an exogenous NO donor, used in their study (1 mM) was too high based on our data, which may have produced harmful effects^21^.

Interestingly, our study revealed that NO produced during conidiation is closely associated with *con* gene expression. In particular, NO scavenging significantly suppressed transcription of *ccg-2*, *con-10* and *con-13,* which are highly expressed in conidiophores^40^. This suggests that NO may regulate conidiophore development by controlling expression of these genes. However, deletion of *con-10* and *con-13* did not exhibit any clear phenotypes during vegetative growth and conidiation in our study (Supplementary Figs S2 and S3). Same observation was already demonstrated in a previous study^41^. It is obscure how NO is involved in controlling conidiation with *con-10* and *con-13* although *ccg-2* encodes an internal component of conidia^42^. A possibility is that NO may be involved in the regulation of circadian rhythm because *ccg-2* and *con-10* are known as clock controlled genes in *Neurospora*^40^,^42^,^43^. As a consequence, conidia development which is controlled by circadian rhythm can be affected by NO. If NO is associated with expression of clock controlled genes, it is possible that level of intracellular NO may be oscillated during a day. Endogenous ROS level is known to follow circadian rhythm in *Neurospora*^44^. However, there is no report for RNS level. In our study, NO was detected during conidiation in conidiophores 16 hours after conidiation induction, but not at 8 or 24 hours, indicating that intracellular NO may be rhythmically changed during a day. Further investigation should be needed for this.

NO may also regulate hyphal growth during submerged culture in VM. Intracellular NO levels were increased at 8–16 hours in culture and decreased after 24 hours, and NO was mostly detected in the cytosol or vacuoles of hyphae. NO production may occur early during hyphal development and is not associated with late stages of vegetative growth. A previous study demonstrated that ROS generated by NADPH oxidase Nox1 is involved in the regulation of hyphal growth in *N. crassa*^45^. However, ROS generated by Nox enzymes controls sexual development in many fungi^46^. Cooperation of ROS (reactive oxygen species) and RNS (reactive nitrogen species) should be considered in the regulation of fungal vegetative growth.

In conclusion, our results demonstrate that NO is endogenously generated during early vegetative growth and conidia formation on conidiophores in *N. crassa*. Generated NO may be involved in the regulation of submerged hyphal growth in liquid and conidia formation (maturation), and transcription of *con-10* and *con-13* is closely associated with intracellular NO levels.

## Methods

### Reagents and materials

Tetraethoxysilane (TEOS) and sodium methoxide (NaOMe) were purchased from Fluka (Buchs, Switzerland). (Aminoethylaminomethyl)phenethyltrimethoxysilane (AEMP3) was purchased from Gelest (Tullytown, PA). Ethanol (EtOH) and ammonia solution (NH_4_OH, 30 wt% in water) were purchased from Fisher Scientific (Fair Lawn, NJ). Nitric oxide (NO, 99.99%) and argon (Ar) gases were obtained from Dong-A Scientific (Seoul, South Korea) or Sung-Kang Special Gas (Seoul, South Korea). Other solvents and chemicals were analytical-reagent grade and used as received. A Millipore Milli-Q UV Gradient A10 System (Bedford, MA) was used to purify distilled water to a final resistivity of 18.2 MΩ·cm and a total organic content of ≤6 ppb.

### Synthesis and characterization of NO-releasing silica nanoparticles

The synthesis and characterization of NO-releasing silica nanoparticles have been described previously^30^. Briefly, an aminoalkoxysilane solution was prepared by dissolving AEMP3 (1.86 mmol) in 20 mL of EtOH and 4 mL of MeOH in the presence of NaOCH_3_ (1.86 mmol). The solution was then placed into 10 mL vials equipped with stir bars. The vials were placed in a Parr bottle, connected to an in-house NO reactor, and flushed with Ar six times to remove O_2_ in the solution. The reaction bottle was pressurized to 10 atm NO for 3 days with continuous stirring of the silane solution. Prior to removing the *N*-diazeniumdiolate-modified AEMP3 silane sample (AEMP3/NO), unreacted NO was purged from the chamber with Ar. The silane solution was prepared by mixing TEOS (2.80 mmol) and AHAP3/NO (1.86 mmol; corresponding to 40 mol%, balance TEOS) in the EtOH/MeOH solution for 2. The silane solution was then added into 22 mL of EtOH and 6 mL of ammonia catalyst (30 wt % in water) and mixed vigorously for 30 min at 4 °C. The precipitated nanoparticles were collected by centrifugation (5000 rpm, 5 min), washed with EtOH several times, dried under ambient conditions for 1 h, and stored in a sealed container at –20 °C until used.

Nitric oxide release from the *N*-diazeniumdiolate-modified AEMP3 silica nanoparticles was measured in deoxygenated PBS (0.01 M, pH 7.4) at 37 °C using a Sievers NOA 280i chemiluminescence NO analyzer (Boulder, CO). Nitric oxide released from the donors was transported to the analyzer by a stream of N_2_ (70 mL·min^−1^) passed through the reaction cell. The instrument was calibrated with air passed through a NO zero filter (0 ppm NO) and a 24.1 ppm NO standard gas (balance N_2_).

The size of NO-releasing silica nanoparticles was characterized by scanning electron microscopy (SEM). To obtain high-quality images, samples were coated with a thin layer of platinum (~5 nm thickness) using a Cressington 108 auto sputter coater (Watford, England). SEM images of AEMP3 silica nanoparticles were collected on a Hitachi S4700 field-emission SEM (Tokyo, Japan) using an accelerating voltage of 10 keV (source working distance of 7.7 mm).

### Fungus and culture condition

Wild type *Neurospora crassa* (ORS-SL6a, mat a) and knockout mutant strains were obtained from the Fungal Genetics Stock Center (FGSC, Manhattan, KS, USA) and conserved as conidial masses in silica gel at −20 °C. Culture of fungal strains from silica stocks was initiated by placing silica gels on VM agar media with (mutants) or without (wild type) hygromycin (200 μg/ml). Agar culture flasks and tubes were incubated at 30 °C in the dark for 2–3 days and then at 25 °C in the light for 11–12 days. For liquid culture, VM liquid inoculated with fungal spores (10^6^/ml) and incubated at 30 °C in the dark with shaking (130 rpm).

### Detection of intracellular nitric oxide (NO)

To detect intracellular NO during hyphal development in liquid media, VM liquid (15 ml) was inoculated with conidia (final concentration of 10^6^ conidia/ml) and incubated at 30 °C with shaking. Fungal mycelia were harvested at each indicated time and washed with 1x phosphate buffered saline (PBS). Fungal mycelia were then resuspended in new PBS. To detect intracellular NO, DAF-FM diacetate (amino-5-methylamino-2′,7′-difluorescein diacetate; Life Technologies, Grand Island, NY, USA) was added to the mycelial suspension to make the final concentration of 20 μM and incubated at room temperature for 1 hour. The suspension was centrifuged at 10,000 rpm for 5 minutes and the supernatant was discarded. Fungal mycelia were washed with 1x PBS three times to remove residual DAF-FM diacetate and resuspended in new PBS. Suspended mycelia were mounted on a glass slide and observed under a fluorescence microscope using a 465–495 nm excitation filter (Nikon, Tokyo, Japan).

Production of intracellular NO was also examined during conidiation which was synchronously induced from the mycelial mat placed on VM agar media. Spores were inoculated into 15 ml VM liquid (10^6^ spores/ml) and incubated at 30 °C for 20 hours with shaking. Fungal mycelia were collected on a filter paper disc (Whatman No. 1, 9 cm in diameter) and dried. The mycelial mat on filter paper was then placed on the surface of VM agar media and incubated at 30 °C in the dark for 3 days. At each indicated time, aliquots of fungal cultures were sampled and suspended in PBS. Intracellular NO was detected using DAF-FM diacetate (final concentration 20 μM) as described earlier.

### Treatment with cPTIO, an NO scavenger

The NO scavenger, cPTIO (2-(4-carboxyphenyl)-4,5-dihydro-4,4,5,5-tetramethy-1 H-imidazolyl-1-oxy-3-oxide; Cayman, Ann Arbor, MI, USA), was used to confirm production of intracellular NO and investigate the effect of NO scavenging on hyphal development and conidiation. VM liquid (15 ml) was inoculated with conidia (final concentration of 10^6 ^conidia/ml) and incubated at 30 °C with shaking. Aliquots of fungal mycelia were harvested at each indicated time and washed with 1x phosphate buffered saline (PBS). Fungal mycelia were then resuspended in new PBS and treated with cPTIO (10 mM final concentration in PBS) for 20 minutes. After treatment with cPTIO, fungal mycelia were washed with PBS three times and intracellular NO was measured using DAF-FM diacetate (20 μM) as described above.

To treat fungi with cPTIO during conidiation, spores were inoculated into 15 ml of VM liquid and incubated at 30 °C for 20 hours with shaking. Fungal mycelia were harvested, dried on a filter paper disc (Whatman No. 1, 9 cm in diameter), and placed on the surface of VM agar media. VM agar plates were incubated at 30 °C. Fungal mycelia were harvested from VM agar plates and suspended in PBS at indicated times. cPTIO was added to mycelia suspensions (10 mM final concentration) and incubated for 20 minutes. After washing with PBS three times, fungal mycelia were resuspended in new PBS and DAF-FM diacetate was added (final concentration 20 μM). Detection of intracellular NO was performed as described previously.

To examine the effects of cPTIO on fungal development, cPTIO was added to VM liquid culture (final concentration 10 mM) or spread on VM agar media. Fungal spores were inoculated (10^6^/ml) in 15 ml of VM liquid containing cPTIO (10 mM) and inoculated culture tubes were incubated at 30 °C with shaking. At indicated times, fungal cultures were sampled and observed under an optical microscope. cPTIO was also spread on VM agar in 60 mm petri dishes (100 μl of 10 mM cPTIO). To determine effects on basal hyphae extension, approximately 10^4^ spores were inoculated in the center of a VM agar plate and the diameter of hyphal extension was measured. After 24 hours, hypha were harvested from the plate and dried, and the hyphal mass was measured. For the effects on conidiation, fungal mycelia collected from liquid culture were spread on the surface of VM agar media covered by cPTIO. Plates were incubated at 30 °C in the dark for 2 days and then at 25 °C in the light. Aerial hyphal development and conidia formation were analyzed using an optical microscope. After 1 week, plates were placed in larger petri-dishes (100 × 40 mm) and 50 ml of sterile water was added to cover culture plates. Petri-dishes were agitated and suspensions were filtered through 2 layers of miracloth (EMD Millipore, Billerica, MA, USA) to remove fungal hypha. Spores in the filtered spore suspensions were counted using a hemacytometer (Marienfeld, Germany).

### Confocal microscopy

To further analyze the production of NO within fungal cells, we examined samples treated with DAF-FM diacetate using confocal microscopy (IX-83, Olympus, Tokyo, Japan). For liquid culture, fungal spores were inoculated in 15 ml of liquid VM (10^6^/ml) and incubated with shaking at 30 °C. For conidiation culture, a fungal mycelial mat was placed on VM agar media and incubated at 30 °C as described previously. At each indicated time during culture, fungal mycelia were sampled and submerged in PBS. Treatment with DAF-FM diacetate was carried out and samples were observed and imaged. Sections in the Z-axis were acquired at 1.5 μm intervals.

### Assay for the effect of exogenous NO on fungal development

Sodium nitroprusside (SNP; Sigma-Aldrich, St. Louis, MO, USA), a chemical that generates NO, and nitric oxide (NO)-releasing AEMP3 silica nanoparticles were used in the treatment with exogenous nitric oxide. NO released in SNP solution at different concentrations was quantitated by measuring the total concentration of nitrite (NO_2_^−^) and nitrate (NO_3_^−^), as NO is quickly oxidized to nitrite and nitrate. Measurements were performed using an NO colorimetric assay kit (Biovision, Milpitas, CA, USA) following the manufacturer’s protocol.

To determine the effects of exogeneous NO on fungal vegetative growth, SNP was added to VM agar media (0.001–0.1 mM) and wild type fungal spores (10^4 ^spores) were inoculated in the center of the plates. After incubation at 30 °C in the dark for 24 hours, the diameter of mycelial growth was measured. AEMP nanoparticles were added in VM liquid media at the concentration of 0–0.1 μg/ml and fungal spores were inoculated (10^4^/ml). Liquid culture flasks were incubated at 30 °C with shaking for 3 days. After fungal mycelia were harvested and dried by filtering the culture through filter paper and vaccuum-drying overnight, the weight of dried mycelia was measured.

To examine the effects of SNP on conidiation, SNP or AEMP nanoparticles were added to 1 ml of VM liquid placed in a glass tube (13 × 10 mm) at 0.001–0.1 mM concentrations. Wild type fungal spores (10^4^) were inoculated on the surface of VM liquid. Tubes were incubated at 30 °C in the dark for 3 days and then moved to 25 °C in the light. Growth of aerial hypha and conidiation were monitored during incubation. After 5 days, 3 ml of water was added into the tubes, which were vigorously shaken. The resulting mixtures were filtered through 2 layers of miracloth to produce conidial suspensions, and conidia were counted using a hemacytometer.

### Quantitative PCR analysis

Expression of putative conidiation related genes (NCU08769, *con-6*; NCU09235, *con-8*; NCU07325, *con-10*; NCU07324, *con-13*; NCU08726, *fluffy*; NCU08457, *ccg-2*; NCU09873, *con-3*; NCU00478, *acon-2*; NCU07617, *acon-3*) was analyzed during formation of aerial hypha and conidia. Fungal mycelia collected from VM liquid culture (as described above) were placed on a filter paper disc (Whatman No. 1, 9 cm in diameter) and dried. The mycelial mat on filter paper was placed on the surface of VM agar media and incubated at 30 °C in the dark. After 8, 16, and 24 hours, aerial hypha and conidia were harvested and total RNA was extracted using the Takara RNAiso Plus kit according to the manufacturer’s protocol (Takara Bio Inc., Shiga, Japan). Total RNA was treated with RNase-free DNase (Toyobo Co., Ltd., Osaka, Japan) at 37 °C for 1 hour to remove genomic DNA, and RNA concentration was measured using a nanodrop (Biotek, Winooski, VT, USA). Equal amount of RNA (16 ng) from all samples was used to synthesize cDNA using the ReverTra Ace^®^ qPCR RT mater mix with gDNA remover kit (Toyobo Co., Ltd., Osaka, Japan). Thermocycle conditions were as follows: 60 min at 37 °C and 5 min at 95 °C. Target genes (10 putative conidiation related genes) were amplified using gene specific primers (Table 2) and quantified at every thermal cycle using iQ SYBR Green Supermix (BioRad, Hercules, CA, USA) and the CFX96^TM^ real time RT-PCR system (Bio-Rad, Hercules, CA, USA). Actin was amplified as a reference gene. The cycle threshold (Ct) was determined and the relative target gene expression level between the sample and the control was calculated as previously described^47^: ratio = 2^−{(Ct target−Ct reference)sample−(Ct target−Ct reference)control}^.

### Statistical analysis

All data were obtained from at least 3 replicate measurements and 2 or 3 replicate experiments. Student’s *t* test was performed to determine significance between data points. Significant differences were established at *p* < 0.05 or *p* < 0.01 (*denotes *p* < 0.05 and **denotes *p* < 0.01).

## Additional Information

**How to cite this article**: Pengkit, A. *et al*. Identification and functional analysis of endogenous nitric oxide in a filamentous fungus. *Sci. Rep.* **6**, 30037; doi: 10.1038/srep30037 (2016).

## Supplementary Material

Supplementary Information

## Figures and Tables

**Table 1:** *N. crassa* candidate genes homologous to NOS of other organisms.

Organisms	Homologous genes in *N. crassa*		
*Homo sapiens*	nNOS	eNOS	iNOS
	aNCU09741 (b5.2E-43)	NCU09741 (3.9E-37)	NCU09741 (1.8E-42)
	NCU05185 (1.1E-35)	NCU05185 (9.1E-24)	NCU09727 (6.4E-30)
	NCU04077 (2.8E-23)	NCU04077 (6.4E-19)	NCU04077 (1.6E-29)
	NCU09727 (2.7E-19)	NCU09727 (2.9E-10)	NCU05185 (1.6E-29)
*Magnaporthe oryzae*	NOL2		NOL3
	NCU05185 (0)		NCU05185 (0)
	NCU05006 (0)		NCU05006 (0)
	NCU08062 (2.4E-21)		NCU09741 (4.9E-27)
	NCU09727 (2.6E-20)		NCU01086 (1.7E-21)
	NCU09741 (8.6E-19)		NCU06327 (7.9E-19)
*Physarum polycephalum*	Physnosa		Physnosb
	NCU09741 (0)		NCU09741 (0)
	NCU05185 (1.4E-45)		NCU05185 (2.8E-45)
	NCU04077 (2.6E-35)		NCU04077 (1.8E-34)
	NCU09727 (2.6E-22)		NCU09727 (1.9E-34)
*Inonotus obliquus*	KM519595		KP052853
	NCU09741 (0)		NCU05185 (0)
	NCU05185 (0)		NCU05006 (0)
	NCU04077 (3.5E-27)		NCU09741 (0)
	NCU09727 (3.4E-25)		NCU01086 (1.1E-24)
	NCU03697 (4.7E-17)		NCU02031 (2.3E-24)
	NCU05238 (1.4E-10)		NCU04077 (1.5E-23)

^a^Homologous gene ID number in *N. crassa* starts with NCU.

^b^Number in parenthesis is expected value.

**Table 2:** Primers used in quantitative PCR analyses of conidiation related genes.

No.	Sequence	Primer details
GP9F	CCAAGGAGCACTCCAAGAAG	NCU08769 (con-6) forward
GP9R	ACACTTTGGGGTTGTTGAGG	NCU08769 (con-6) reverse
GP11F	GGGCTTGATGGATCAAAA	NCU09235 (con-8) forward
GP11R	ATCCCACATCTTGGCAAT	NCU09235 (con-8) reverse
GP12F	AGGAAGAGGTTCAGGCCATC	NCU07325 (con-10) forward
GP12R	GGCTCAAAGCTGCCGCTGGA	NCU07325 (con-10) reverse
GP13F	AAGACTGGAAGGATACCGTT	NCU07324 (con-13) forward
GP13R	GATTGACCATACAGCCGACA	NCU07324 (con-13) reverse
GP14F	TCCAGCATCTCGTTGTCATC	NCU08726 (fluffy) forward
GP14R	GGTGAAAAACGGGAGGAAAT	NCU08726 (fluffy) reverse
GP15F	TTACTGCTGCCAGTCTATGT	NCU08457 (ccg-2) forward
GP15R	TAACATCGTCCTTGCAGCAC	NCU08457 (ccg-2) reverse
GP16F	CCATCGAGTACATCCCCAAC	NCU09873 (con-3) forward
GP16R	GCCGGAGATAAAGTGGAACA	NCU09873 (con-3) reverse
GP17F	AGAAGCCTTCACCCTTCTCC	NCU00478 (acon-2) forward
GP17R	CAGTAGCTGGTGCTGTGGAA	NCU00478 (acon-2) reverse
GP18F	GGATCAGGCGTTGAAGAAAA	NCU07617 (acon-3) forward
GP18R	TAGCCCGTTGAAGATTGGTC	NCU07617 (acon-3) reverse
