# Supplementary material for: Identification and functional analysis of endogenous nitric oxide in a filamentous fungus
Source: Sci Rep. 2016 Jul 18;6:30037. doi: 10.1038/srep30037 (PMC4948021; doi:10.1038/srep30037)
Supplement: Supplementary Information [file srep30037-s1.pdf]

Supplementary Information

Identification and functional analysis of endogenous nitric oxide in a filamentous fungus

Anchalee Pengkit[1], Seong Sil Jeon[2], Soo Ji Son[3], Jae Ho Shin[3], Ku Yeon Baik[1,2], Eun Ha Choi[1,2], and Gyungsoon Park[1,2*]

[1] Plasma Bioscience Research Center, Kwangwoon University, Seoul, 01897, Republic of Korea

[2] Department of Electrical and Biological Physics, Kwangwoon University, Seoul, 01897, Republic of Korea

[3] Department of Chemistry, Kwangwoon University, Seoul, 01897, Republic of Korea

* To whom correspondence should be addressed. E-mail: gyungp@kw.ac.kr

Methods

Analysis for responses of knockout mutants to exogeneous nitric oxide (NO)

Knockout mutants of transcription factors and serine/threonine protein kinases that are defective in vegetative hyphal growth and conidiation [1,2] (see supplementary table S1) were examined for responses to exogenous NO. Fungal spores were harvested from 2 weeks old culture tubes. Sterile water (5-6 ml) was added in culture tubes and tubes were vigorously shaken. The fungal suspension was filtered through 2 layers of miracloth and filtrated suspension was centrifuged at 5,000 rpm for 5 min. Spore pellet was resuspended in new sterile water. For examining vegetative hyphal growth, spore suspension (1 µl; 10^4 spores) was inoculated on the center of VM agar plate on which 100 µl of 0.2 mM SNP (sublethal dose to wild type) was spread. Then, plates were incubated at 30 °C in the dark for 24 h and diameter of radial hyphal growth was measured. For examining conidiation, spore suspension (1 µl; 10^4 spores) was spread on the surface of VM liquid (2 ml) in tube and the tubes were incubated at 25 °C in the light. After 1 and 3 days, height of aerial hypha was measured. After 5 days, conidia were collected in 10 ml water and number was counted using hemacytometer. All measurements were performed in 3 replicates and significance of data difference between wild type and mutants was examined by Student's *t* test. Screening was repeated 4 times, and mutants showing recovery were selected in every screening.

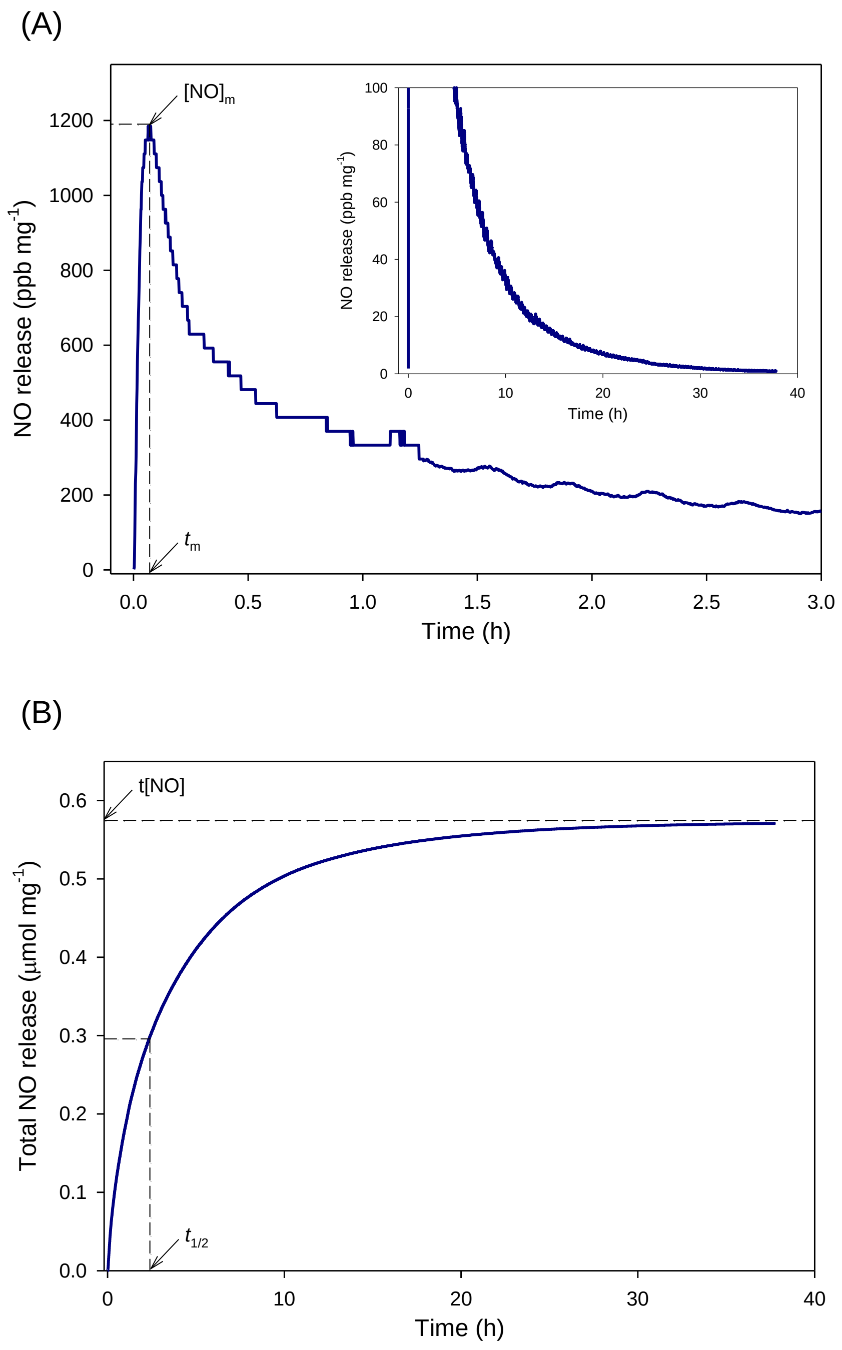

Supplementary Figure S1. Real-time NO release profiles (A) and total NO release amount (B) for 40 mol% *N*-diazeniumdiolated AEMP3 silica nanoparticles in deoxygenated PBS (0.1 M, pH 7.4) at 37°C. Inset plot represents the expansion of graph (A) at 100 ppb·mg^{-1} NO release.

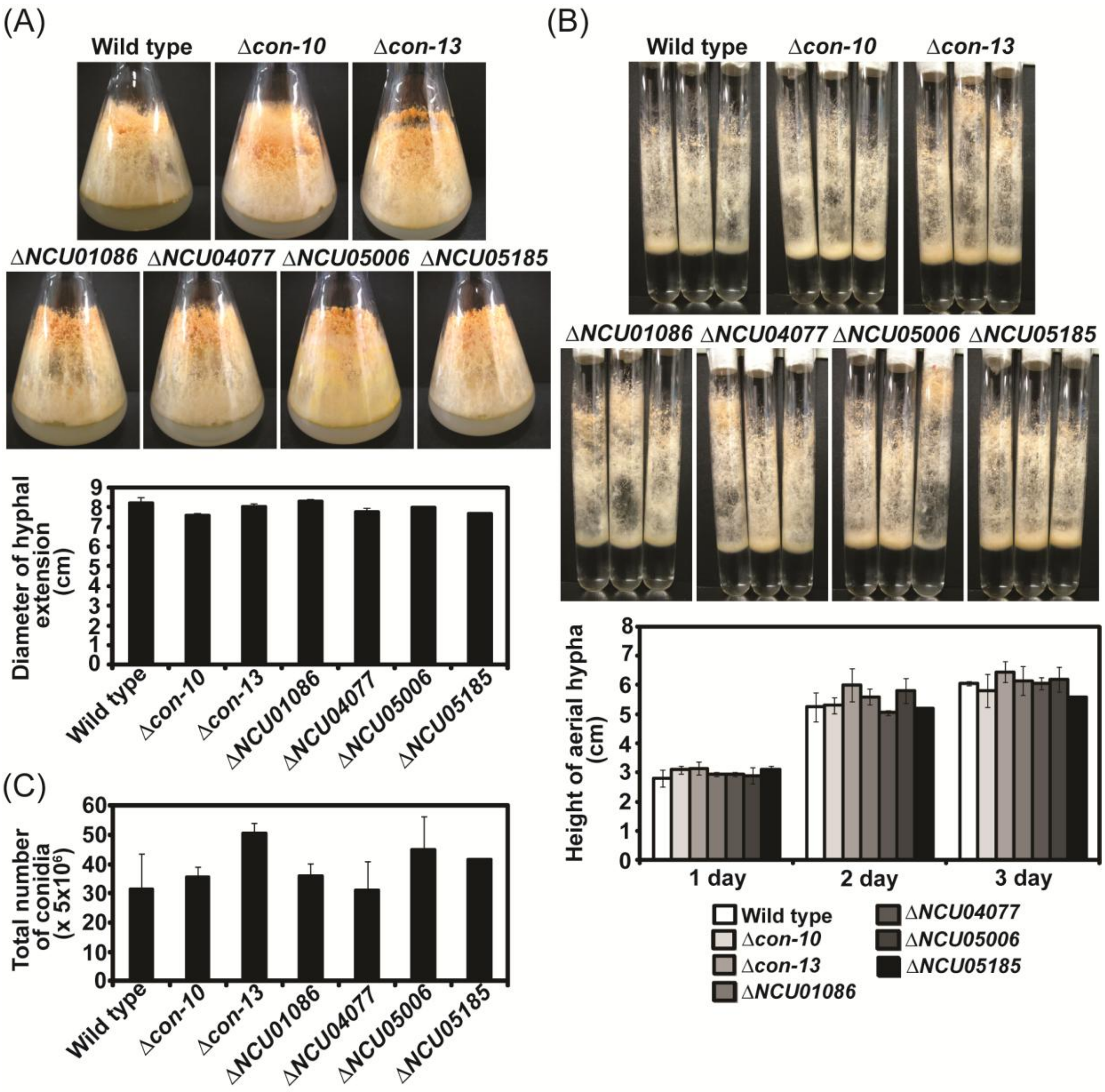

Supplementary Figure S2. Vegetative growth and asexual development of *Neurospora* mutants. (A) Wild type (ORS-SL6a) and knockout mutants grown in VM agar flask for 5 days (pictures), and basal hyphal extension on VM agar plate incubated for 24 h (graph). (B) Aerial hyphal growth of wild type and knockout mutants on the surface of VM liquid. Pictures were taken after incubation for 3 days. (C) Total number of conidia harvested after 5 days. Inoculation, harvest, and measurement were carried out following procedures described in Methods. All measurements were performed in 3 replicates.

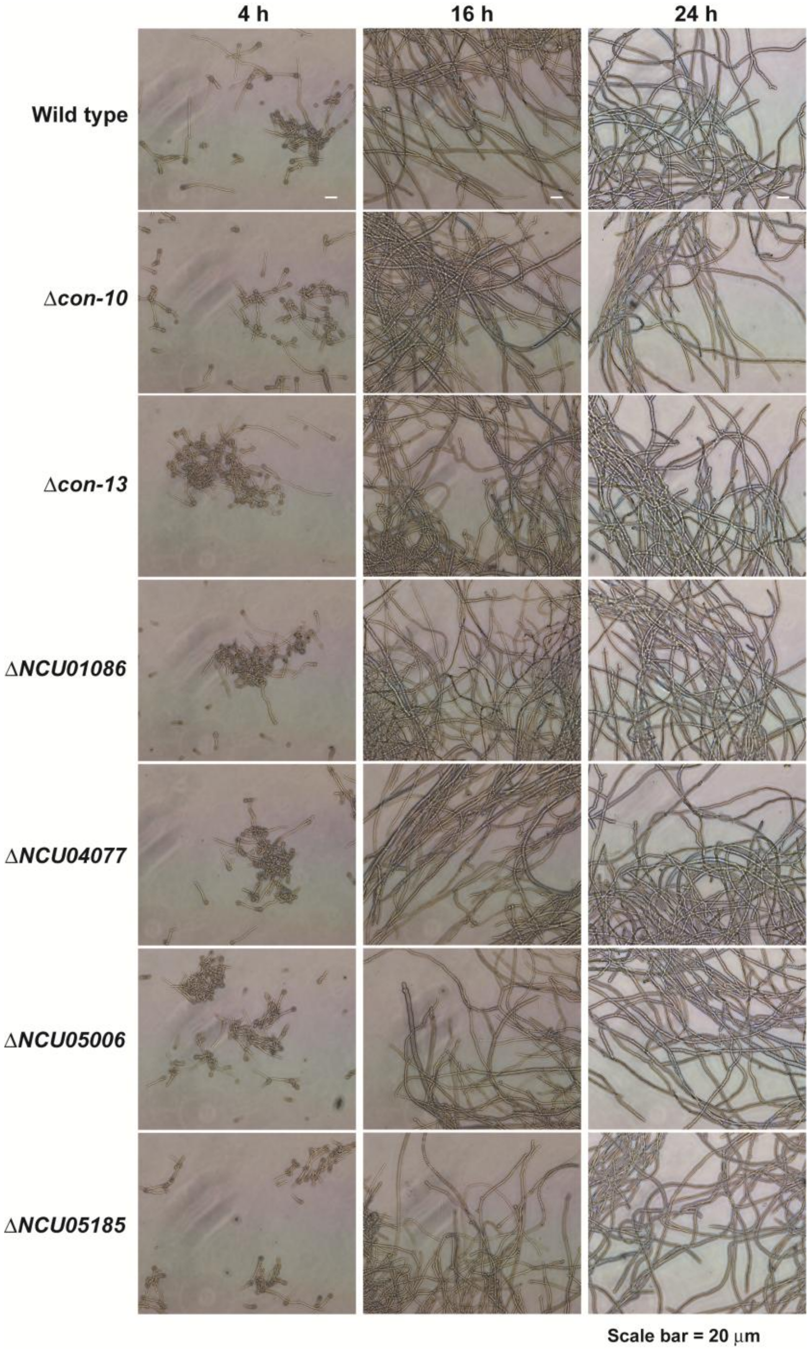

Supplementary Figure S3. Germination and hyphal development of wild type and knockout mutants in VM liquid.

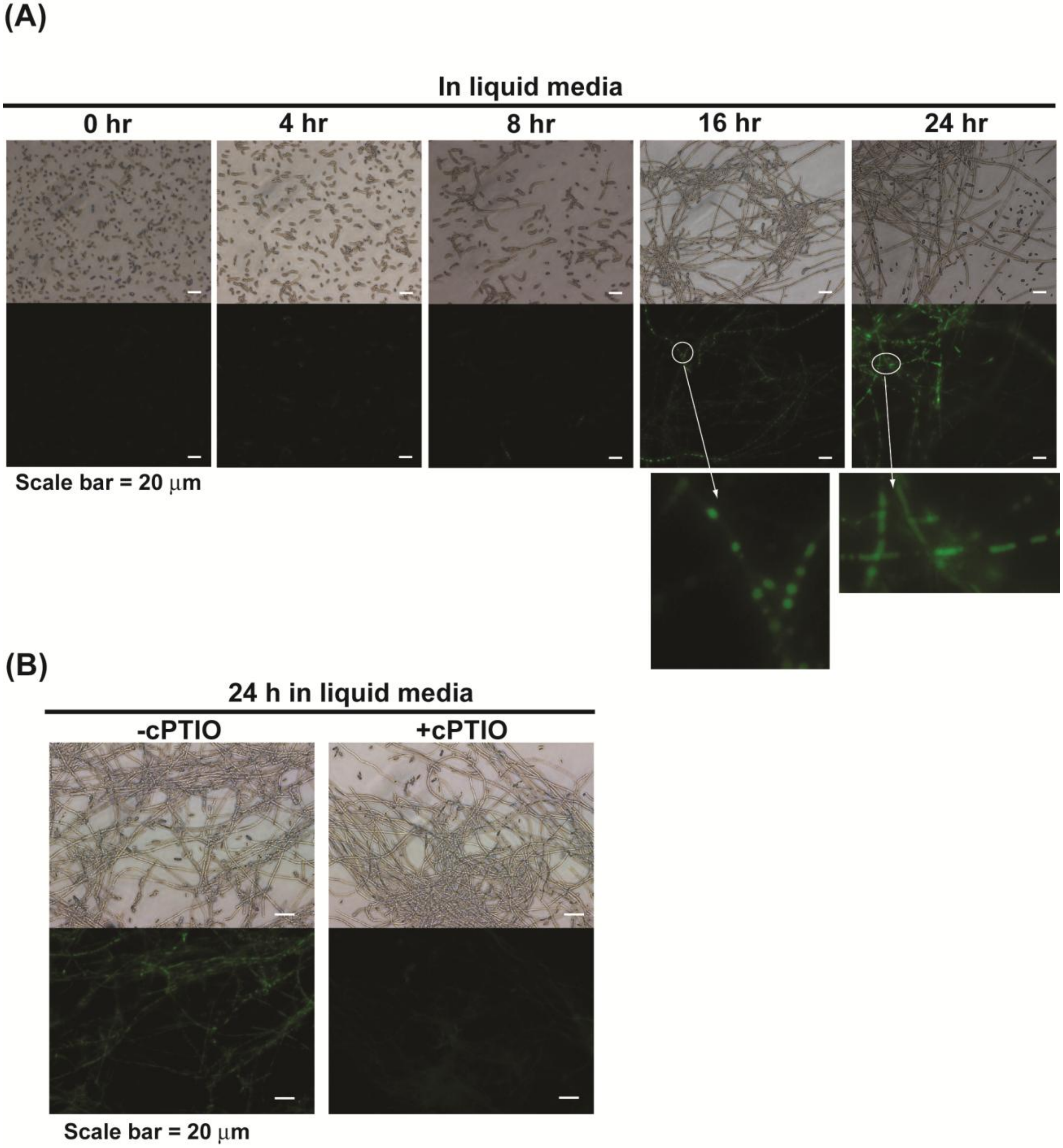

Supplementary Figure S4. (A) Intracellular NO detected in *Fusarium oxysporum f.sp. lycopersici* using DAF-FM diacetate during microconidia formation in VM liquid, (B) Intracellular NO detected in 24 h liquid culture with or without 50 mM cPTIO. Top and bottom of each picture indicate images captured in optical and fluorescent (465-495 nm excitation filter) mode, respectively. NO detection and treatment with cPTIO in *F. oxysporum* were performed as described in methods.

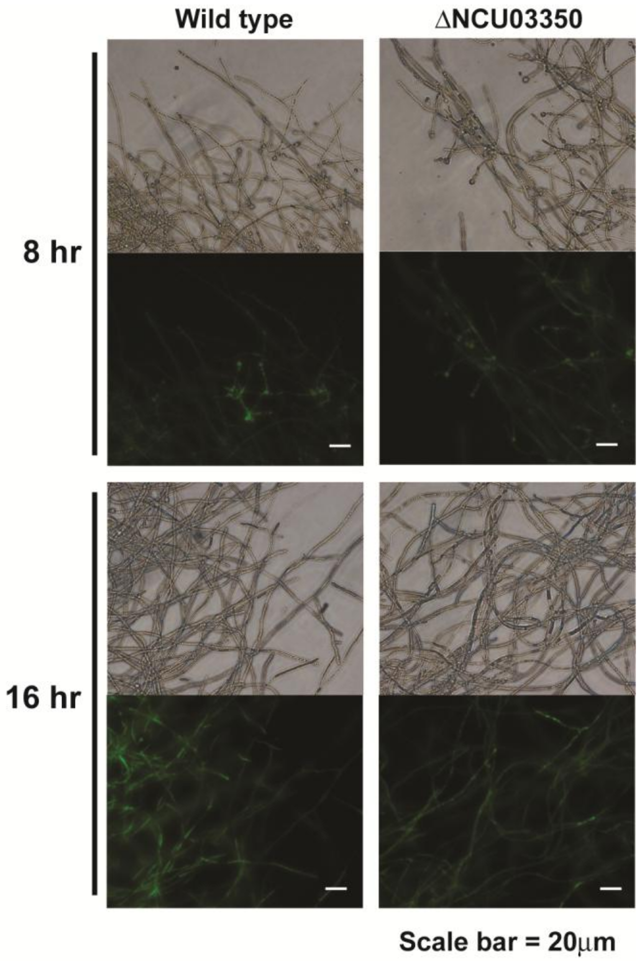

Supplementary Figure S5. Intracellular NO detected using DAF-FM diacetate in *N. crassa* wild type and knockout mutant of a putative xanthine oxidase gene (NCU03350) during vegetative growth in submerged VM culture. NO was detected after 8 and 16 h. Top and bottom of each picture indicate images captured in optical and fluorescent (465-495 nm excitation filter) mode, respectively.

Supplementary Table S1. List of *N. crassa* knockout mutants used in the study

FGSC #	Genotype	FGSC #	Genotype
TF[1]	NCU00289::hyg+	Kinase[2]	NCU00188::hyg+
TF	NCU00329::hyg+	Kinase	NCU00406::hyg+
TF	NCU00499::hyg+	Kinase	NCU00587::hyg+, microconidia
TF	NCU00902::hyg+	Kinase	NCU00914::hyg+
TF	NCU00945::hyg+	Kinase	NCU01187::hyg+, heterokaryon
TF	NCU02017::hyg+	Kinase	NCU01498::hyg+
TF	NCU02094::hyg+	Kinase	NCU01797::hyg+
TF	NCU02214::hyg+	Kinase	NCU01940::hyg+
TF	NCU02896::hyg+	Kinase	NCU02234::hyg+
TF	NCU03320::hyg+	Kinase	NCU02393::hyg+
TF	NCU03489::hyg+	Kinase	NCU03071::hyg+
TF	NCU03593::hyg+	Kinase	NCU03124::hyg+
TF	NCU03686::hyg+	Kinase	NCU03200::hyg+
TF	NCU03931::hyg+	Kinase	NCU03523::hyg+
TF	NCU04179::hyg+	Kinase	NCU03571::hyg+
TF	NCU04390::hyg+	Kinase	NCU03894::hyg+
TF	NCU04731::hyg+	Kinase	NCU04096::hyg+
TF	NCU04866::hyg+	Kinase	NCU04426::hyg+
TF	NCU05051::hyg+	Kinase	NCU04612::hyg+
TF	NCU05383::hyg+	Kinase	NCU04747::hyg+
TF	NCU06068::hyg+	Kinase	NCU04990::hyg+
TF	NCU06407::hyg+	Kinase	NCU05655::hyg+
TF	NCU06411::hyg+	Kinase	NCU05658::hyg+
TF	NCU06656::hyg+	Kinase	NCU06182::hyg+
TF	NCU06799::hyg+	Kinase	NCU06202::hyg+
TF	NCU07139::hyg+	Kinase	NCU06240::hyg+
TF	NCU07392::hyg+	Kinase	NCU06638::hyg+
TF	NCU07535::hyg+	Kinase	NCU06685::hyg+
TF	NCU07788::hyg+	Kinase	NCU07024::hyg+
TF	NCU07945::hyg+	Kinase	NCU07296::hyg+
TF	NCU08294::hyg+	Kinase	NCU07378::hyg+
TF	NCU08651::hyg+	Kinase	NCU07399::hyg+
TF	NCU08726::hyg+	Kinase	NCU07872::hyg+
TF	NCU09205::hyg+	Kinase	NCU09071::hyg+
TF	NCU09739::hyg+	Kinase	NCU09123::hyg+

1, Transcription Factor

2, Serine/Threonine Protein Kinase

Supplementary Table S2. Conidiation of knockout mutant *ΔNCU05658* after treatment with SNP

Height of aerial hypha (cm)

	Wild type			*ΔNCU05658*		
	without SNP	with SNP	% SNP *	without SNP	with SNP	% SNP
1 day	1.3 ± 0.3	1.0 ± 0.1	80.0 ± 17.6	0.6 ± 0.1	0.7 ± 0.0	118.9 ± 20.1
3 days	1.8 ± 0.3	1.9 ± 0.0	109.1 ± 16.1	0.9 ± 0.1	1.0 ± 0.2	114.5 ± 4.8

Number of spores (x 10^6 / ml)

	Wild type			*ΔNCU05658*		
	without SNP	with SNP	% SNP	without SNP	with SNP	% SNP
5 days	104.3 ± 19.7	98.0 ± 16.0	94.4 ± 8.6	37.3 ± 14.4	44.3 ± 8.5	129.6 ± 45.3

* % SNP = (value of with SNP / value of without SNP) x 100

References

1. Colot, H. V. *et al*. A high-throughput gene knockout procedure for *Neurospora* reveals functions for multiple transcription factors. *Proc. Natl. Acad. Sci. U S A* **103**, 10352-10357 (2006).
2. Park, G. *et al*. Global analysis of serine-threonine protein kinase genes in *Neurospora crassa*. *Eukaryot. Cell* **10**, 1553-1564 (2011).
